# Feasibility of Symptom monitoring WIth Feedback Trial (SWIFT) for adults on hemodialysis: a registry-based cluster randomized pilot trial

**DOI:** 10.1186/s12882-023-03399-5

**Published:** 2023-11-22

**Authors:** Neeru Agarwal, Karan K. Shah, Kathryn Dansie, Paul N. Bennett, Lavern Greenham, Chris Brown, Brendan Smyth, Stephen McDonald, Shilpanjali Jesudason, Andrea K. Viecelli, Rachael L. Morton, on behalf of the Symptom monitoring With Feedback Trial (SWIFT) Investigators, Carmel Hawley, David W. Johnson, David Harris, Lilliana Laranjo, Cecile Couchoud, Fergus J. Caskey, Suetonia Palmer, Matthew Jose, R. John Simes, Braden Manns, William Handke, Enrico Coiera, Rebecca Mister, Portia Westall

**Affiliations:** 1 NHMRC Clinical Trials Centre, University of Sydney, Level 6, Medical Foundation Building, 92-94 Parramatta Rd, Camperdown, NSW 2050 Australia; 2 Australia and New Zealand Dialysis and Transplant Registry, South Australian Health and Medical Research Institute, Adelaide, Australia; 3 Medical and Clinical Affairs, Satellite Healthcare, San Jose, USA; 4 Clinical & Health Sciences, University of South Australia, Adelaide, Australia; 5 Department of Renal Medicine, St George Hospital, Kogarah, Australia; 6 Faculty of Health and Medical Sciences, University of Adelaide, Adelaide, Australia; 7 Central Northern Adelaide Renal and Transplantation Service, Adelaide, Australia; 8 Department of Nephrology, Princess Alexandra Hospital, Brisbane, Australia; 9 Faculty of Medicine, University of Queensland, Brisbane, Australia

**Keywords:** Hemodialysis, Patient Reported Outcome Measures, Quality of Life, Symptom Assessment, Registries, Cluster analysis, Randomized Controlled Trial, Medical Record Linkage

## Abstract

**Background:**

Patients with kidney failure on hemodialysis (HD) experience considerable symptom burden and poor health-related quality of life (HRQoL). There is limited use of patient reported outcome measures (PROMs) in facility HD units to direct immediate care, with response rates in other studies between 36 to 70%. The aim of this pilot study was to evaluate feasibility of electronic PROMs (e-PROMs) in HD participants, with feedback 3-monthly to the participants’ treating team, for severe or worsening symptoms as identified by the Integrated Palliative Outcome Scale (IPOS-Renal), with linkage to the Australian and New Zealand Dialysis and Transplant (ANZDATA) registry, compared with usual care.

**Methods:**

This is a registry-based cluster-randomized controlled pilot trial involving all adults receiving HD in 4 satellite units in Australia over a 6-month period. HD units were cluster randomized 1:1 to the control (HRQoL data collection only) or intervention arm (symptom monitoring with feedback to treating team every 3 months). Feasibility was assessed by participant response rate (percentage of eligible HD participants, including new incident participants, who completed the questionnaire at each time point); retention rate (percentage of participants who completed the baseline questionnaire and all subsequent measures); and completion time. HRQoL and symptom burden scores are described.

**Results:**

There were 226 unique participants who completed the e-PROMs (mean age 62 years, 69% males, 78% White-European, median dialysis vintage 1.62 years). At 6 months, response rate and retention rate for the intervention arm were 54% and 68%, respectively, and 89% and 97% in the control arm. Median time to complete IPOS-Renal was 6.6 min (5.3, 10.1) at 3 months, and when combined with the outcome measure (EQ-5D-5L), the median time was 9.4 min (6.9, 13.6) at 6 months.

**Conclusions:**

Electronic symptom monitoring among HD participants with feedback to clinicians is feasible. Variations in response and retention rates could be potentially explained by the lengthier questionnaire, and higher frequency of data collection time points for participants in the intervention arm. A definitive national RCT is underway.

**Trial registration:**

ACTRN12618001976279 (07/12/2018).

**Supplementary Information:**

The online version contains supplementary material available at 10.1186/s12882-023-03399-5.

## Background

Patients with kidney failure experience considerable symptom burden [1, 2]. This is associated with poor health related quality of life (HRQoL) [3], and independently predicts hospitalization and mortality [4]. Underreporting of symptoms by patients [5, 6] frequently results in underappreciation of potentially treatable symptoms [7]. Patient reported outcome measures (PROMs) are standardized questionnaires that assess how patients feel and function as reported by the patients themselves [8]. They facilitate communication between the patient and their healthcare providers about their symptoms and functional status, reveal issues that may otherwise not have been identified, and may improve quality of life [9]. National kidney registries have recently initiated the routine collection of PROMs, thereby embedding this in clinical practice. The Dutch [10], Swedish [11]and Scottish [12] kidney registries suggest routine collection of PROMs is feasible and they can identify underrecognized and treatable symptoms. However, there are challenges, such as low response rates, organizational barriers, and variable motivation of both patients and healthcare professionals that need to be considered when introducing PROMs into routine care [13].

Currently, there is limited use of PROMs in facility hemodialysis (HD) units in Australia and New Zealand to direct immediate care [14]. In this cluster randomized pilot study, the primary aim was to evaluate the feasibility of electronic PROMs (e-PROMs) in HD participants, with 3-monthly feedback to the participants’ treating team for severe or worsening symptoms, compared with usual care, in an Australian context. This study builds on the qualitative interviews and analysis among patients, clinicians, and nurses regarding acceptability of e-PROMs which has been reported elsewhere [15, 16].

## Methods

### Study design

Four Australian publicly funded metropolitan and rural HD units of varying size and sociodemographics were cluster randomized 1:1 to the control (HRQoL e-PROM collection only) or intervention arm (symptom monitoring e-PROM with feedback to treating team every 3 months) (Supplementary Item S1). This study design was selected to minimize treatment contamination by clinical staff within and across HD units. All adults aged ≥ 18 years undergoing maintenance HD were eligible. Prevalent participants at baseline were enrolled. At each subsequent time point, new incident participants were assessed for eligibility and added because the primary aim of the study was to assess the feasibility of implementation of e-PROMs into HD units.

### Control arm

HD units randomized to the control arm continued with usual care, and completed the HRQoL outcome measure, EuroQOL 5 dimensions 5 levels (EQ-5D-5L) (Supplement Item S2) at baseline and 6 months. The EQ-5D-5L consists of 5 dimensions assessing participants’ health state (mobility, self-care, usual activities, pain/discomfort, and anxiety/depression) scored on a 5-level scale from 1 (no problems) to 5 (extreme problems). Scores for each dimension were combined into a 5-digit number describing the participant’s health profile, which was converted into a utility index with a maximum value of 1 (indicating full and perfect health) using value sets elicited from the general population [17]. In addition, a visual analogue scale (VAS) recorded the participants self-rated health status “today” ranging from 0 (worst perceived health state) to 100 (best perceived health state). EQ-5D-5L has the highest amount of complete data collected amongst utility-based HRQoL questionnaires in patients with kidney failure, making it the best option for repeated measures collection [3].

### Intervention arm

Units randomized to the intervention arm completed the Integrated Palliative Outcomes Scale-Renal (IPOS-Renal, patient version, one week recall) questionnaire (Supplement Item S3) at baseline, 3 and 6 months, in addition to the EQ-5D-5L at baseline and at 6 months. The IPOS-Renal questionnaire has been validated in patients with advanced kidney disease in an Australian population [18] and is the most familiar PROMs across kidney services in Australia and New Zealand [14]. It measures 15 common physical symptoms affecting people with kidney failure on a 5-point Likert scale from 0 (not at all affected) to 4 (overwhelmingly affected), and integrates psychological, spiritual, communication and practical concerns. Each symptom is self-reported according to severity “over the past week”. The total score reflects symptom burden.

Following administration of the questionnaire at baseline, 3 and 6 months, a tailored email (Supplement Item S4) was sent to each treating nephrologist and nurse unit manager in the intervention arm. Participants who reported IPOS-Renal symptom scores of 3 or 4, indicating severe or overwhelming symptoms, were highlighted in the body of the email. The email also contained hyperlinks to evidence-based symptom management guidelines, and clinicians were encouraged to discuss symptoms with their study participants at the next outpatient visit.

### Procedure

The administration and data collection process for this study is reported in detail elsewhere [16]. Briefly, e-PROM responses were collected for both arms using Qualtrics software via a tablet (Samsung Galaxy Tab A V.10.5) at the participant’s routine HD session either before, during or after HD. Nurses at each unit were responsible for distributing the tablet. Individualized participant quick response (QR) codes linked the participant responses to the Australian and New Zealand Dialysis and Transplant registry (ANZDATA) records. The e-PROMs data were collected within a two-week period, stored briefly on a secure cloud-based platform, and periodically transferred to secure servers with linked data to the ANZDATA registry. Clinical and demographic variables for all participants were extracted from ANZDATA.

### Pilot trial outcomes

Feasibility of the intervention was assessed by participant response rate, retention rate, and questionnaire completion time. Secondary outcomes included the need for assistance to complete the e-PROMs (intervention arm only), and exploratory outcomes included HRQoL and symptom burden.

### Randomization

Initially six HD units (clusters) were randomized 1:1 by a statistician to the control or intervention arm. Sites were grouped according to locally connected clusters, and clusters were paired to maximize the balance between groups. All clusters were simultaneously allocated in pairs to the 2 study arms (permuted blocks of length 2). To determine the order of randomization, sites were stratified within each Australian state by metropolitan/rural status, public/private sector, prior use of PROMs, and size of site. Allocation was concealed from sites until site initiation. Of note, two private sector HD units that were randomized did not have local governance and research contract approvals in time for study commencement and were excluded. This did not affect stratification by other factors.

### Sample size

With a sample size of 438 participants from 6 sites, we were able to estimate a 70% participation rate to within 95% confidence interval of ± 5%. We expected 288 participants from the four sites that participated.

### Statistical analysis

Baseline characteristics were reported using descriptive statistics by frequencies (n, %), means (SD), and medians (interquartile range [IQR]), as appropriate. A completed questionnaire was defined as containing responses to each of the 15 symptoms on the IPOS-Renal and all five dimensions in the EQ-5D-5L. A ‘complete case’ was defined as a participant who completed the baseline questionnaire and all subsequent questionnaires as per study allocation.

Response rates were assessed cross sectionally at baseline, 3- and 6- months. This was defined as the total number of participants who completed the questionnaire, out of all the eligible participants in the unit at that time point, including new incident participants. Retention rate at 6 months was calculated to assess the proportion of participants that continued in the study from baseline. The denominator did not include new incident participants and was the total number of participants that completed the questionnaire at baseline but had not died, transferred to another unit, nor changed modality (e.g., received kidney transplant, changed to peritoneal dialysis), to reflect “true retention”. In a similar fashion, we assessed the retention rate at 3-months for participants in the intervention arm. For purposes of assessing completion time, we excluded completed responses that took > 60 minutes because this likely reflected a connectivity fault with submission of the e-PROMs, but in all other relevant analyses these participants were included. The proportion of participants in the intervention arm that required assistance to complete the questionnaire from nurses or friend/relative was also reported.

Furthermore, change in e-PROM scores were described for ‘complete cases’. We did not perform hypothesis testing as this study was not powered to detect a difference between the groups. The frequency of each health state and proportion of participants who reported ‘some’ level of problem (i.e. levels 2 or more) for each dimension were calculated. Changes in EQ-5D-5L health states were analyzed using a Paretian classification method wherein participants were classified as “same imperfect health” if they reported the same health state at both time points, “improved health state” if there was improvement in any of the dimensions with no deterioration in the remaining ones, “worse health state” if there was worsening in any of the dimensions with no deterioration in the remaining ones, or “mixed health state” if there was both improvement and worsening in any of the dimensions [19]. In addition, the EQ-5D-5L utility index and the EQ-VAS score were reported as means (SD). A 0.07 (7%) change in the utility index was considered clinically meaningful [20]. IPOS-Renal questionnaire was completed only in the intervention arm and individual item scores, a total physical symptom score and an overall score were reported as means (SD). A total physical symptom score was calculated as the sum of the 15 physical symptom scores (maximum score 60). The overall IPOS-Renal score was calculated as the sum of scores from questions 2 to 9 (maximum score 88) [21]. Statistical analyses were performed using R (Version 4.1.1). This study was reported according to CONSORT 2010 checklist for pilot or feasibility trials [22].

### Ethical considerations

This study was approved by the Central Adelaide Local Health Network Human Research Ethics Committee (CALHN HREC/18/CALHN/481). Informed consent was taken from participants prior to participation in the study. They were provided with written information about the study and had the opportunity to opt-out. All methods were carried out in accordance with relevant guidelines and regulations.

## Results

**Fig. 1:**
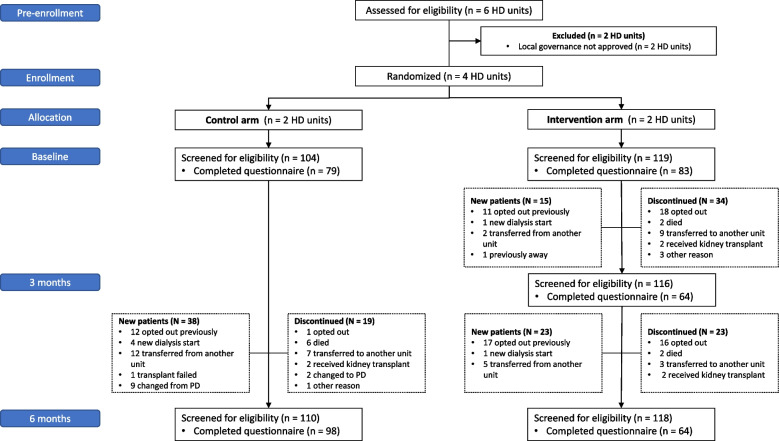
Study consort diagram for all participants. HD, hemodialysis; PD, peritoneal dialysis

**Fig. 2:**
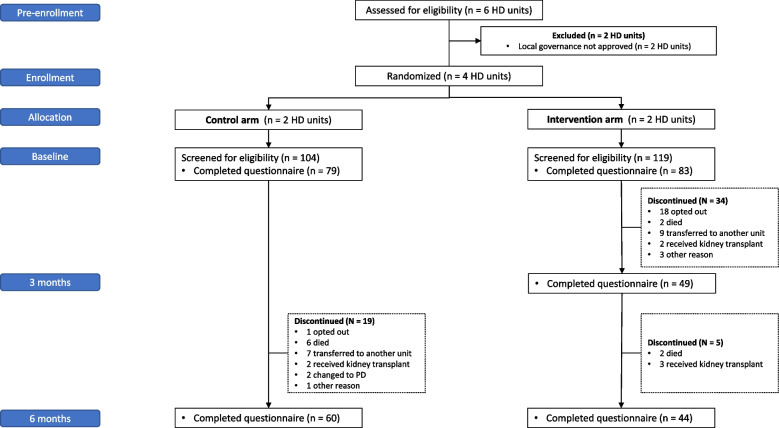
Study consort diagram for ‘complete cases’ (participants who completed baseline and all subsequent questionnaires). HD, hemodialysis; PD, peritoneal dialysis

The SWIFT pilot ran from August 2019 to March 2020. In total, there were 226 unique participants who completed the e-PROMs across both arms with a mean age of 62 years, and majority were male (69%), White-European (78%), with a median HD vintage of 1.62 years (Table 1). Baseline characteristics for participants who completed the baseline questionnaire and all subsequent questionnaires as per study allocation (‘complete cases’) are presented in Supplement Item S5. The movement of participants in and out of the study is further illustrated in Fig. 1, and for ‘complete cases’ in Fig. 2.

**Table 1:** Characteristics of all unique pilot study participants

Variable	All participants (*n* = 226)	Control (*n* = 117)	Intervention (*n* = 109)
Age (years), mean (SD)	62 (15)	61 (16)	63 (12)
Sex			
• Males, n (%)	156 (69)	76 (65)	80 (73)
• Females, n (%)	70 (31)	41(35)	29 (27)
Ethnicity, n (%)			
• White European	177 (78)	81 (69)	96 (88)
• Indigenous	14 (6)	6 (5)	8 (7)
• Other	35 (16)	30 (26)	5 (5)
Co-morbidities^a^, n (%)			
• Diabetes mellitus	111 (49)	56 (48)	57 (52)
• Ischemic heart disease	74 (33)	41 (35)	33 (30)
• Cerebrovascular disease	26 (12)	15 (13)	11 (10)
• Peripheral vascular disease	47 (21)	25 (21)	22 (20)
• Chronic lung disease	42 (19)	22 (19)	20 (18)
Primary kidney disease, n (%)			
• Diabetic Nephropathy	73 (32)	38 (32)	35 (32)
• Glomerulonephritis	53 (24)	23 (20)	30 (28)
• Hypertension	27 (12)	16 (14)	11 (10)
• Other	73 (32)	40 (34)	33 (30)
Years on hemodialysis, median (IQR)	1.62 (0.62, 2.87)	1.56 (0.45, 3.14)	1.71 (0.93, 2.87)

*IQR* Interquartile range, *SD* Standard deviation

^a^Participants were likely to have more than one co-morbidity

### Primary outcomes: response rate, retention rate and completion time

**Fig. 3:**
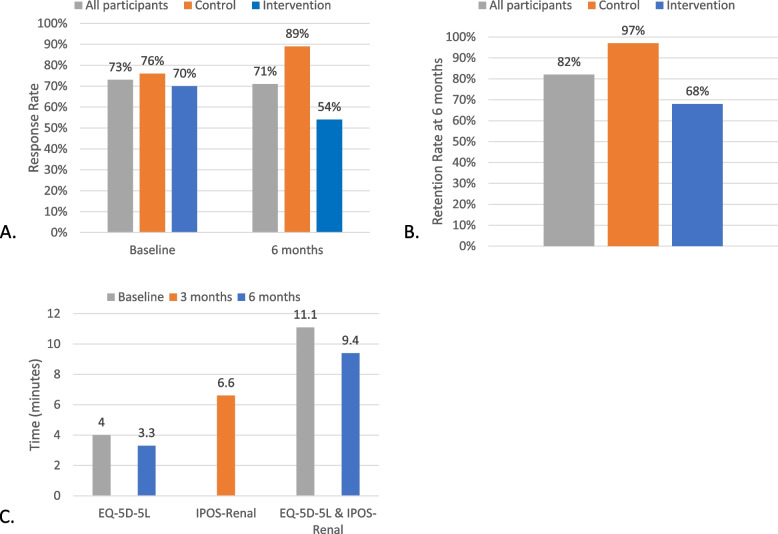
SWIFT pilot primary outcomes **A**. Response rates at baseline and 6 months **B**. Retention rate at 6 months **C**. Completion time

Response rates were assessed overall and by study allocation for all eligible HD participants in the unit, including new incident participants, at baseline and 6-months (Fig. 3A). At baseline, 162 of the 223 possible questionnaires were completed, constituting an overall response rate of 73% (control arm 76% [79/104], intervention arm 70% [83/119]). At 6 months the overall response rate was 71% with 162 of 228 possible questionnaires completed (control arm 89% [98/110], intervention arm 54% [64/118]). In addition, the response rate in the intervention arm at 3-months was 55% (64/116).

Retention rate at 6 months (Fig. 3B) was assessed to determine proportion of participants that remained in the study after completing the baseline questionnaire. In the control arm, 60 of the 79 participants who completed the EQ-5D-5L questionnaire at baseline also completed it at 6-months; but, 17 participants had transferred, changed modality, or died, resulting in a true retention rate of 97% (60/62). In the intervention arm, 49 of 83 participants who completed both the IPOS-Renal and EQ-5D-5L questionnaire at baseline also completed it at 3 months; but of these 13 participants had transferred, changed modality, or died, constituting a true retention rate of 70% (49/70). By 6-months, no additional participant opted out, but 5 participants changed modality or died resulting in a true retention rate of 68% (44/65). Hence at the end of 6 months, the retention rate in the control arm was 97% compared to 68% in the intervention arm.

Time taken to complete the e-PROMs was another feasibility metric (Fig. 3C). Seven completed responses that took longer than 60 minutes (accounting for 1.8% of all completed responses) were excluded in this analysis. The median time to complete the IPOS-Renal at 3 months (*n* = 62) was 6.6 min (IQR 5.3, 10.1). When combined with the outcome measure (EQ-5D-5L), the median completion time at baseline (*n* = 82) was 11.1 min (8.0, 14.3) and 9.4 min (6.9, 13.6) at 6-months (*n* = 62). Further, the median (IQR) time to complete the EQ-5D-5L only in the control arm at baseline (*n* = 78) was 4 min (3.0, 5.6) and 3.3 min (2.5, 4.5) at 6-months (*n* = 97).

### Secondary outcomes: assistance to complete e-PROMs

Fifty-five percent of participants in the intervention arm required some support to complete the e-PROMs. Assistance was mainly provided by a member of nursing staff (95%), and the remaining 5% of assistance was provided by a friend or relative.

### Exploratory outcomes: description of quality of life and symptom burden

Exploratory outcomes are described for the 44 ‘complete cases’ in the intervention arm and 60  ‘complete cases’ in the control arm, i.e., those who completed the baseline and all subsequent measures (Fig. 2, Supplementary Item S5). With regards to quality of life, the mean EQ-5D-5L utility index for the intervention arm increased by 0.10 (0.59 [SD 0.31] at baseline to 0.69 [SD 0.24] at 6-months), and 0.04 in the control arm (0.63 [SD 0.27] at baseline to 0.67 [SD 0.27] at 6-months). In contrast, the mean EQ-VAS score increased by 10 points for the intervention (61 [SD 26] at baseline to 71 [SD 19] at 6-months) and decreased by 1 point in the control arm (74 [SD 21] at baseline to 73 [SD 19] at 6-months).

**Fig. 4:**
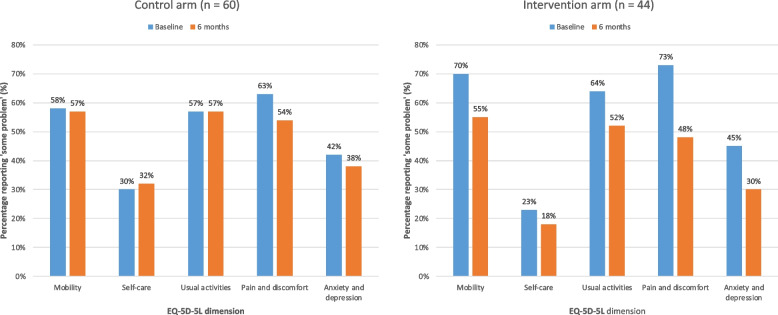
Proportion of participants reporting ‘some’ level of problem (levels 2–5) in the EQ-5D-5L questionnaire by study allocation at baseline and 6-months

**Fig. 5:**
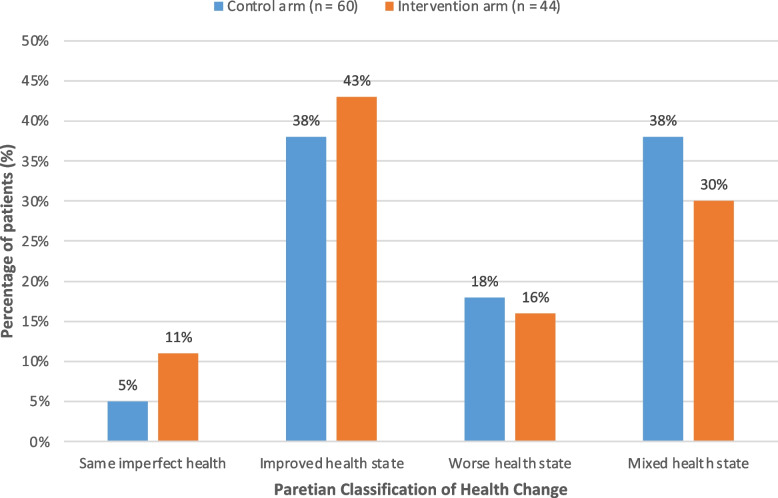
Changes in the health state according to the Paretian Classification of Health Change by study allocation at 6-months

Moreover, Table 2 shows the unique health states observed in participants undergoing HD, with only a handful of observations accounted for by profile 11111 (no problems in any dimension) and none with the worst possible health state (profile 55555). Figure 4 compares the proportions of participants who reported ‘some problem’ (levels 2 to 5) in each of the 5 dimensions at baseline and 6-months for the control and intervention arms. At baseline across both arms, more than 55% of HD participants experienced some difficulty with mobility, usual activities and pain or discomfort; over 40% of participants reported some problem with anxiety and depression; and over 20% reported some problems with self-care. After 6-months, the proportions reporting ‘some problem’ decreased for all five dimensions of the EQ-5D-5L for those in the intervention arm. In the control arm this was seen only for pain or discomfort, and anxiety and depression, with minimal to no reduction in mobility and usual activities, and an increase in the dimension of self-care (Supplement Item S6). Figure 5 shows the distribution of changes according to the Paretian classification of Health Change for the two arms. Among the intervention arm, 43% showed overall improvement, 16% showed overall worsening, 30% showed mixed change and 11% showed no change. The corresponding figures for the control arm were 38% improvement, 18% worsening, 38% mixed change and 5% no change.

**Table 2:** Prevalence of self-reported health states for ‘complete cases’, by study allocation at baseline and 6-months

	**Control (*n* = 60)**				**Intervention (*n* = 44)**			
	**Baseline**		**6 months**		**Baseline**		**6 months**	
**Health state**^**a**^		**n (%)**		**n (%)**		**n (%)**		**n (%)**
**Most frequent**	11121	8 (13)	11111	13 (22)	11111	5 (11)	11111	6 (14)
	11111	5 (8.3)	11121	3 (5)	21121	3 (6.8)	11121	5 (11)
	11112	3 (5)	11221	2 (3.3)	11221	2 (4.5)	21211	4 (9.1)
	21121	2 (3.3)	21311	2 (3.3)	21221	2 (4.5)	11112	2 (4.5)
	21221	2 (3.3)	31111	2 (3.3)	31222	2 (4.5)	11211	2 (4.5)
	31311	2 (3.3)						
	33333	2 (3.3)						
**Worst possible**	55555	0 (0)	55555	0 (0)	55555	0 (0)	55555	0 (0)
**Unique**	…	36 (60)	…	38 (63.3)	…	30 (68.2)	…	25 (56.8)

^a^Health state is 5-digit number that combines the scores for the five dimensions in the EQ-5D-5L i.e., 21311 profile means a 2 (slight problems) for mobility; 1 (no problems) for self-care, 3 (moderate problems) for usual activities, 1 (no problems) for pain/discomfort, 1 (no problems) for anxiety/depression

**Fig. 6:**
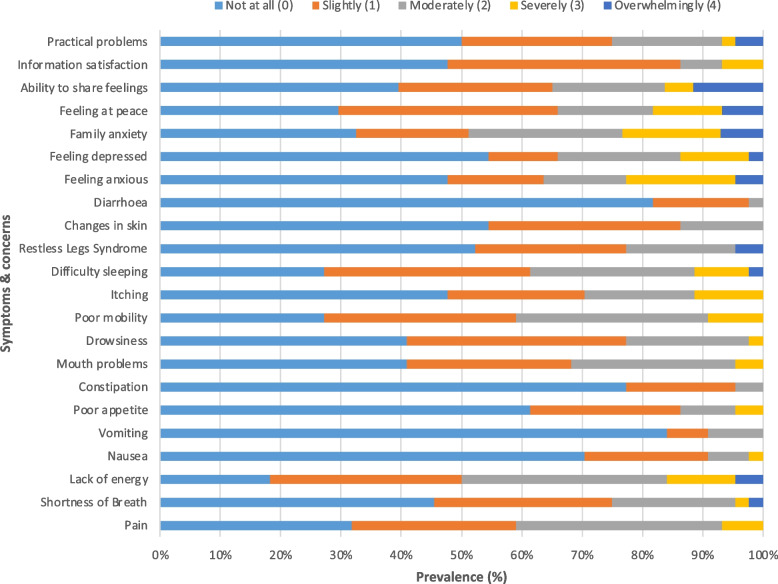
Severity of IPOS-Renal physical and emotional symptoms at baseline

Symptom burden in HD participants was further explored for those in the intervention arm. At baseline the mean overall IPOS-Renal score was 19.6 (SD 12.6) and the mean total physical symptom score was 11.8 (SD 7.1) (Table 3). The five most reported physical symptoms at baseline were lack of energy (50%), poor mobility (41%), pain (41%), difficulty sleeping (39%) and mouth problems (32%). Feeling depressed was reported by 34% of participants, while personal anxiety and family anxiety were reported by 36% and 49%, respectively. Participants experienced a median of 8 (4.8, 10) of 15 physical symptoms at baseline. Around 5% of participants experienced at least 1 physical symptom they classified as severe or overwhelming. Severity of baseline symptoms and concerns are shown in Fig. 6. After 6-months, there were improvements in the mean overall IPOS-Renal score (19.6 vs 15.9) and mean total physical symptom score (11.8 vs 9.3). There were improvements in mean severity scores for most individual symptoms and patient concerns, except for constipation, restless legs syndrome, feeling at peace and practical problems (Table 3). Of note, there was limited improvement in the prevalence of family anxiety (49% vs 46%) which was the most prevalent burden.

**Table 3:** Prevalence and symptom burden scores for ‘complete cases’ in intervention arm at baseline and 6-months

	**Baseline** ***(n* = 44)**		**6 months (*n* = 44)**	
	Mean (SD)	Prevalence^a^ (%)	Mean (SD)	Prevalence^a^ (%)
***Physical symptoms***				
Pain	1.16 (0.96)	41	0.86 (0.90)	30
Shortness of breath	0.86 (0.98)	25	0.66 (0.83)	18
Lack of Energy	1.52 (1.07)	50	1.11 (0.87)	34
Nausea	0.41 (0.73)	9	0.32 (0.67)	11
Vomiting	0.25 (0.62)	9	0.18 (0.54)	8
Poor appetite	0.57 (0.85)	14	0.32 (0.67)	7
Constipation	0.27 (0.54)	5	0.30 (0.59)	7
Mouth problems	0.95 (0.94)	32	0.68 (0.83)	14
Drowsiness	0.84 (0.83)	23	0.66 (0.81)	2
Poor mobility	1.23 (0.96)	41	1.05 (0.86)	25
Itching	0.93 (1.07)	30	0.68 (0.86)	16
Difficulty sleeping	1.25 (1.04)	39	1.14 (1.09)	32
Restless Legs Syndrome	0.80 (1.05)	23	0.86 (0.98)	20
Changes in skin	0.59 (0.73)	14	0.36 (0.53)	2
Diarrhea	0.20 (0.46)	2	0.16 (0.43)	2
***Emotional symptoms***				
Feeling anxious	1.2 (1.3)	36	0.77 (1.11)^b^	23
Feeling depressed	0.95 (1.20)	34	0.44 (0.84)^d^	9
***Other concerns***				
Family anxiety	1.47 (1.30)^b^	49	1.39 (1.32)^d^	46
Feeling at peace	1.30 (1.21)	34	1.30 (1.21)^b^	30
Ability to share feelings	1.23 (1.34)^b^	35	1.19 (1.52)^c^	29
Information satisfaction	0.73 (0.87)	14	0.43 (0.50)^c^	0
Practical problems	0.86 (1.09)	25	0.93 (1.45)^b^	19
**Mean physical symptom score**	**11.8 (7.1)**	**-**	**9.3 (5.5)**	**-**
**Mean overall IPOS score**	**19.6 (12.6)**^**c**^	-	**15.9 (9.1)**^**d**^	-

^a^Prevalence was defined as the proportion of IPOS symptom reported as moderate, severe or overwhelming (scores 2–4). Higher scores indicate greater symptom severity

^b^1 participant was not included in this analysis as they did not complete this question

^c^2 participants were not included in this analysis as they did not complete (part of) this question

^d^3 participants were not included in this analysis as they did not complete (part of) this question

## Discussion

This cluster randomized pilot study is the first Australian trial embedded in a registry database that explores the feasibility of e-PROMs data capture with feedback to the treating team in a HD population. We were able to demonstrate successful deployment of e-PROMs presented on tablet computers, with use of QR reader codes to correctly verify patients from ANZDATA and link the relevant questionnaire for the participant’s allocation and study timepoint.

In this study, the overall response rate of 71% was high when compared with other international studies (36% to 70%) [13], however there was variability among the study arms with a decrease in participant engagement over time. There are potential reasons that may explain these results. First, the shorter questionnaire and less frequent data collection time points in the control arm may have resulted in higher response rates and subsequent better retention rates compared with the somewhat lengthier and more frequently administered questionnaires in the intervention arm. Second, higher response and retention rates in the control arm may have been linked to a greater number of motivated healthcare professionals in those HD units at the time of data collection. Factors such as the absence of key healthcare professionals, a change in focus to core duties during the Christmas holiday period, and the beginning of the COVID-19 pandemic may have impacted staffing resource allocation in the intervention arm, especially at the 3-month time point, thereby resulting in subsequent poor response and retention rates. This finding highlights the impact of real world scenarios and the importance of professional engagement and staff awareness in the use of e-PROMs [12, 23], which has also been suggested by the qualitative interviews from participants in the SWIFT pilot study [15].

With regards to HRQoL and symptom scores, our cohort is similar to those reported in other Australian studies of populations receiving HD [24–26] reinforcing that these participants have reduced HRQoL and high symptom burden. Although this study was not powered to assess the effectiveness of the feedback intervention, participants in the intervention arm reported an important clinical difference of 0.1 (10%) change in the utility on the EQ-5D-5L scale, where a difference of 0.07 (7%) is considered clinically meaningful [20]. Our preliminary results also showed improvement in HRQoL dimensions, EQ-VAS score, and IPOS-Renal scores after the intervention, but this will be addressed in the definitive clinical trial.

This study has several limitations. First, this was a pilot study and inferences about effectiveness of the intervention cannot be made. Second, while the primary purpose of this study was to assess the feasibility of conducting e-PROMs, the fact that new, incident participants were recruited at each time point may have artificially increased the response rate as people may have been more willing to complete a questionnaire initially rather than repeatedly. Third, two private HD sites were unable to obtain local governance permission in time for the pilot, a finding that has been factored in the larger trial to ensure participation from all sectors of HD providers as private units don’t routinely participate in research. Fourth, we do not know the level of nursing support that was received by participants in the control arm, however 55% of participants in the intervention arm required some level of support, which may have included helping patients navigate the survey and overcome technological issues on the tablet, helping patients with impaired vision or dexterity, and arranging additional support via an interpreter or Aboriginal Liaison Officer if needed [15]. This however can limit feasibility and participant engagement given an already stretched workforce [14], especially as nurses have to “fit” e-PROMs collection with existing clinical duties [15]. For the definitive trial, to facilitate participant recruitment at both a site and patient level several strategies have been developed including site support research personnel to assist with data collection and on-site training, translation of trial documents in 7 languages, finger-sign consent on tablets, and a ‘nurse champion’ in each HD unit to increase professional and participant involvement. Further details, including target sample size calculations, are described in the main trial protocol [27]. Fifth, whilst HD units were randomized and stratified based on location, size, and prior use of PROMs, there were imbalances in baseline demographics such as sex and ethnicity between the arms, which may have affected reporting of symptom outcomes [28], although sex is not associated with low response rate [29].

## Conclusion

This Australian cluster-randomized pilot trial demonstrated it is feasible to conduct e-PROM data capture and feedback in routine clinical practice, with good response rates. Our data support the commencement of the definitive trial (ACTRN12620001061921) [27] and provides a framework for national registry-collection of patient-reported outcomes, among patients with kidney failure in Australia.

### Supplementary Information

**Additional file 1.** 

## Data Availability

The datasets generated and analysed during the current study are not publicly available, but requests for access to the SWIFT pilot data can be made to the Principal Investigator, Prof Rachael Morton (rachael.morton@sydney.edu.au), and will be considered by the SWIFT steering committee.

## Abbreviations

ANZDATA: Australian and New Zealand Dialysis and Transplant registry
e-PROMs: Electronic patient reported outcome measures
EQ-5D-5L: EuroQOL 5 dimensions 5 levels
HD: Hemodialysis
HRQoL: Health related quality of life
IPOS-Renal: Integrated Palliative Outcomes Scale-Renal
IQR: Interquartile range
PD: Peritoneal dialysis
PROMs: Patient reported outcome measures
QR: Quick response
SD: Standard deviations
VAS: Visual analogue scale
