# Supplementary material for: Feasibility of Symptom monitoring WIth Feedback Trial (SWIFT) for adults on hemodialysis: a registry-based cluster randomized pilot trial
Source: BMC Nephrol. 2023 Nov 22;24:345. doi: 10.1186/s12882-023-03399-5 (PMC10666372; doi:10.1186/s12882-023-03399-5)
Supplement: Supplementary file 1 — Additional file 1. [file 12882_2023_3399_MOESM1_ESM.docx]

**SWIFT PILOT SUPPLEMENTARY MATERIAL**

**Item S1.** Study schema for the Symptom Monitoring With Feedback Trial (SWIFT).

**Item S2.** The EQ-5D-5L questionnaire.

**Item S3.** The IPOS-Renal (patient version, one week recall) questionnaire.

**Item S4.** Example feedback email**.**

**Item S5.** Characteristics of ‘complete cases’ (participants who completed all questionnaires) by study allocation.

**Item S6.** Frequency of levels by dimensions for ‘some problems’ in the EQ-5D-5L questionnaire by study allocation at baseline and 6-months.

**Item S1.** Study schema for the Symptom Monitoring With Feedback Trial (SWIFT).

**
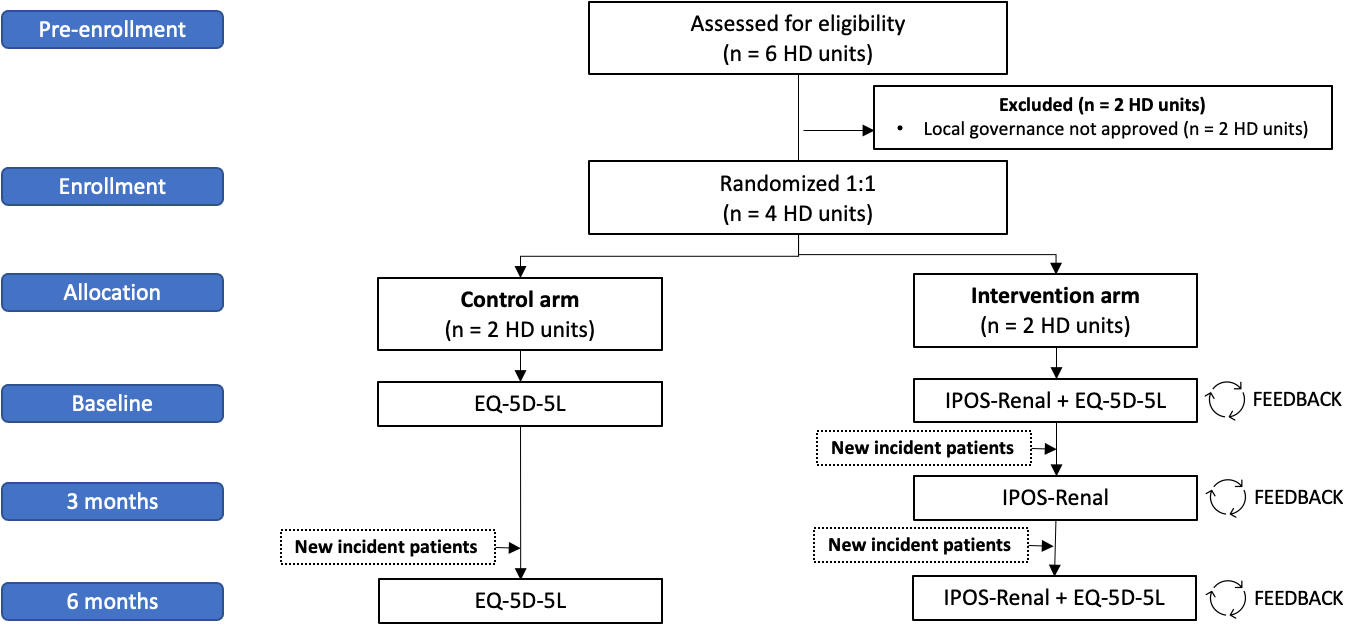
**

Abbreviations: EQ-5D-5L, EuroQOL 5 Dimensions, 5 Levels instrument; HD, haemodialysis; IPOS-Renal, Integrated Palliative Outcome Scale questionnaire.

**Item S2.** The EQ-5D-5L questionnaire.


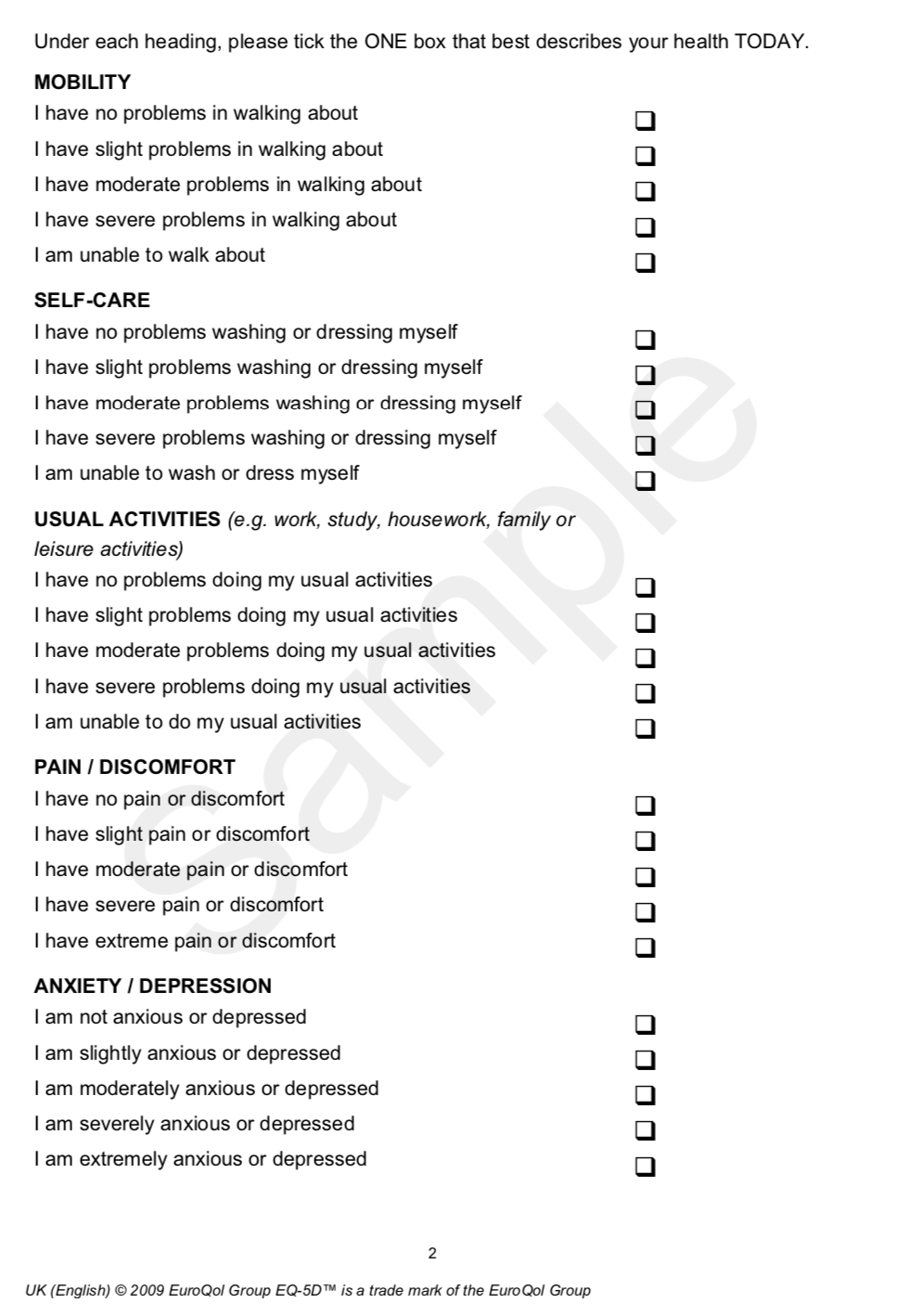


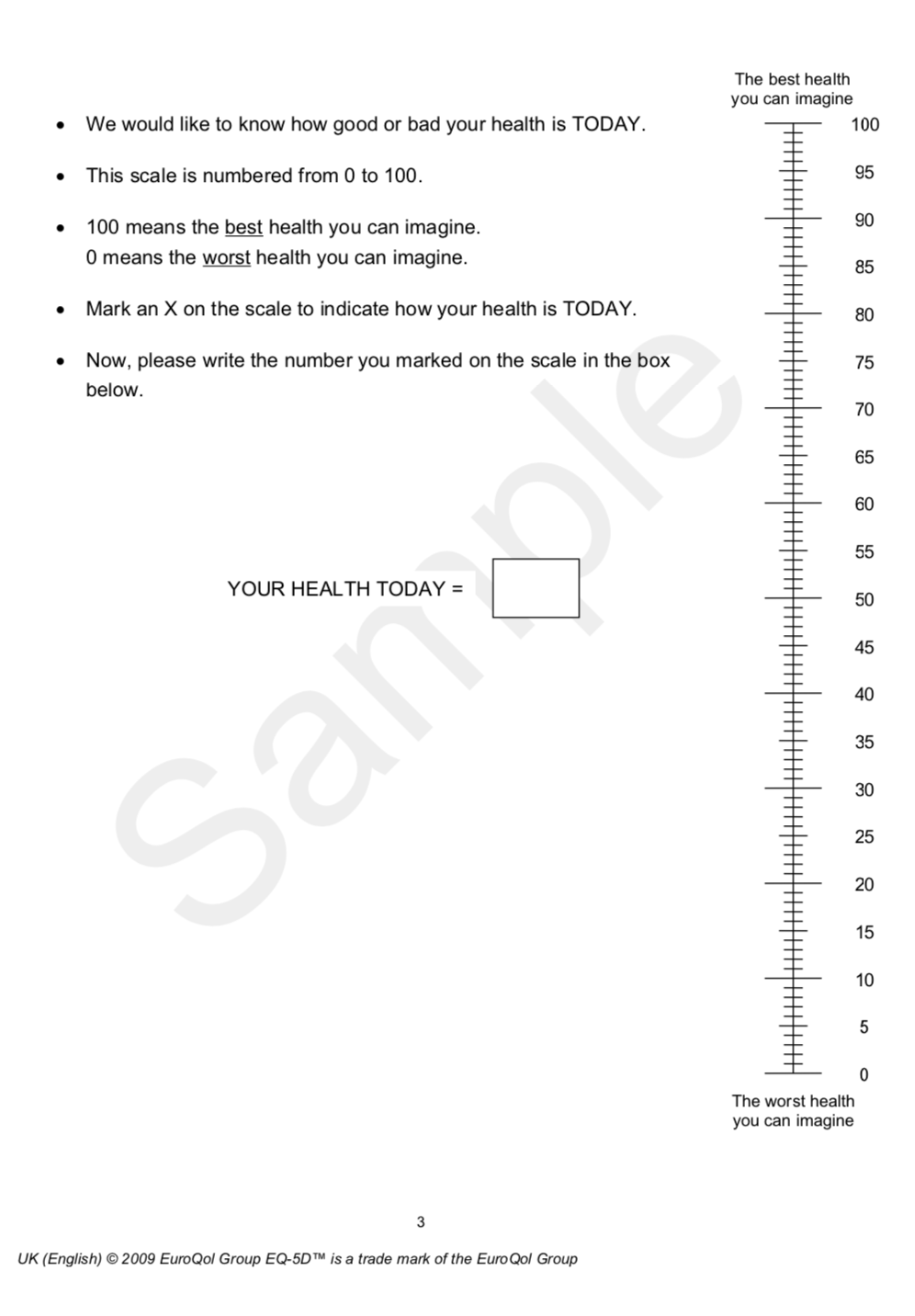


**Item S3.** The IPOS-Renal (patient version, one week recall) questionnaire. **
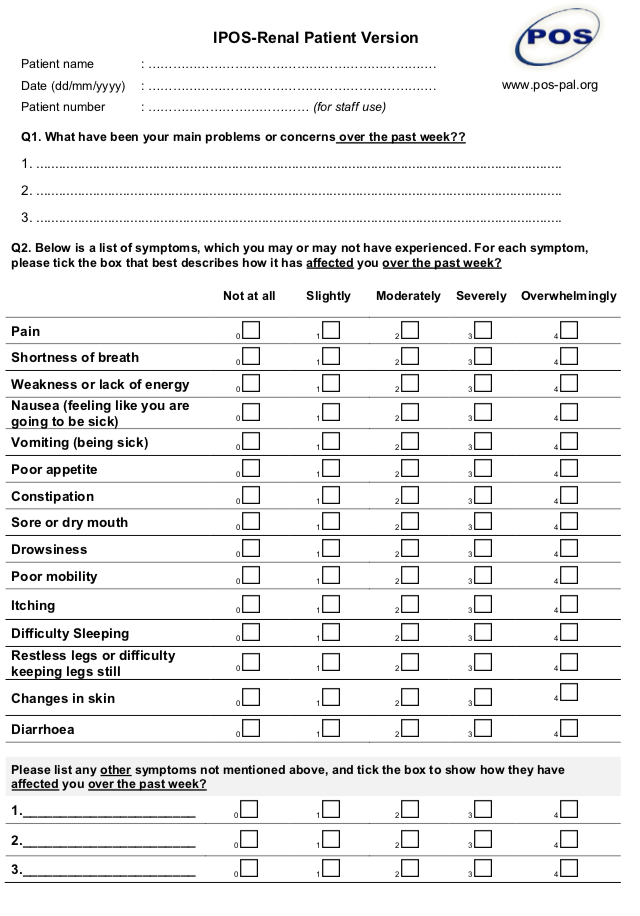
**


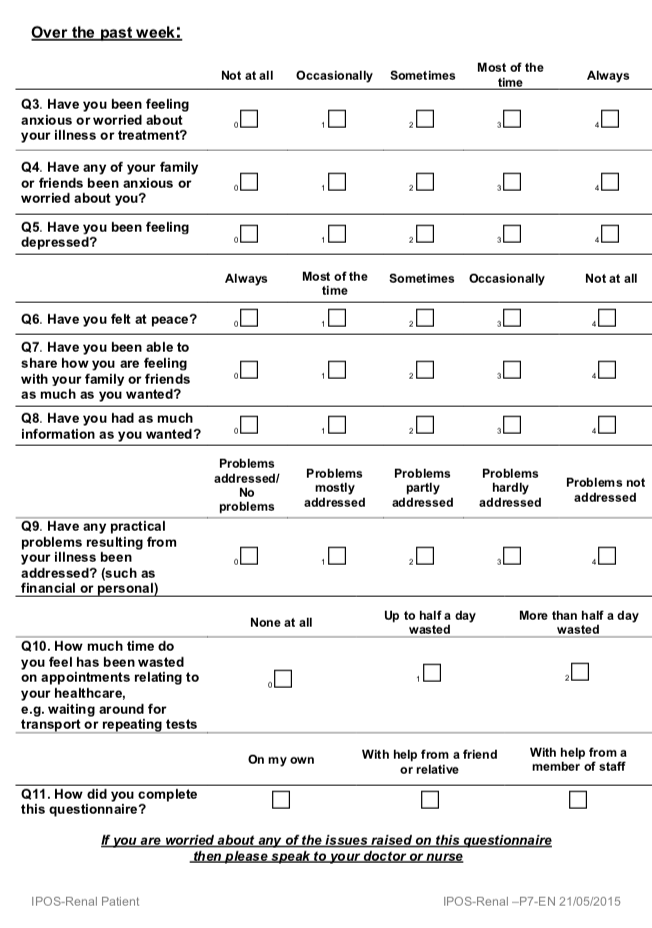


**Item S4.** Example feedback email.

**
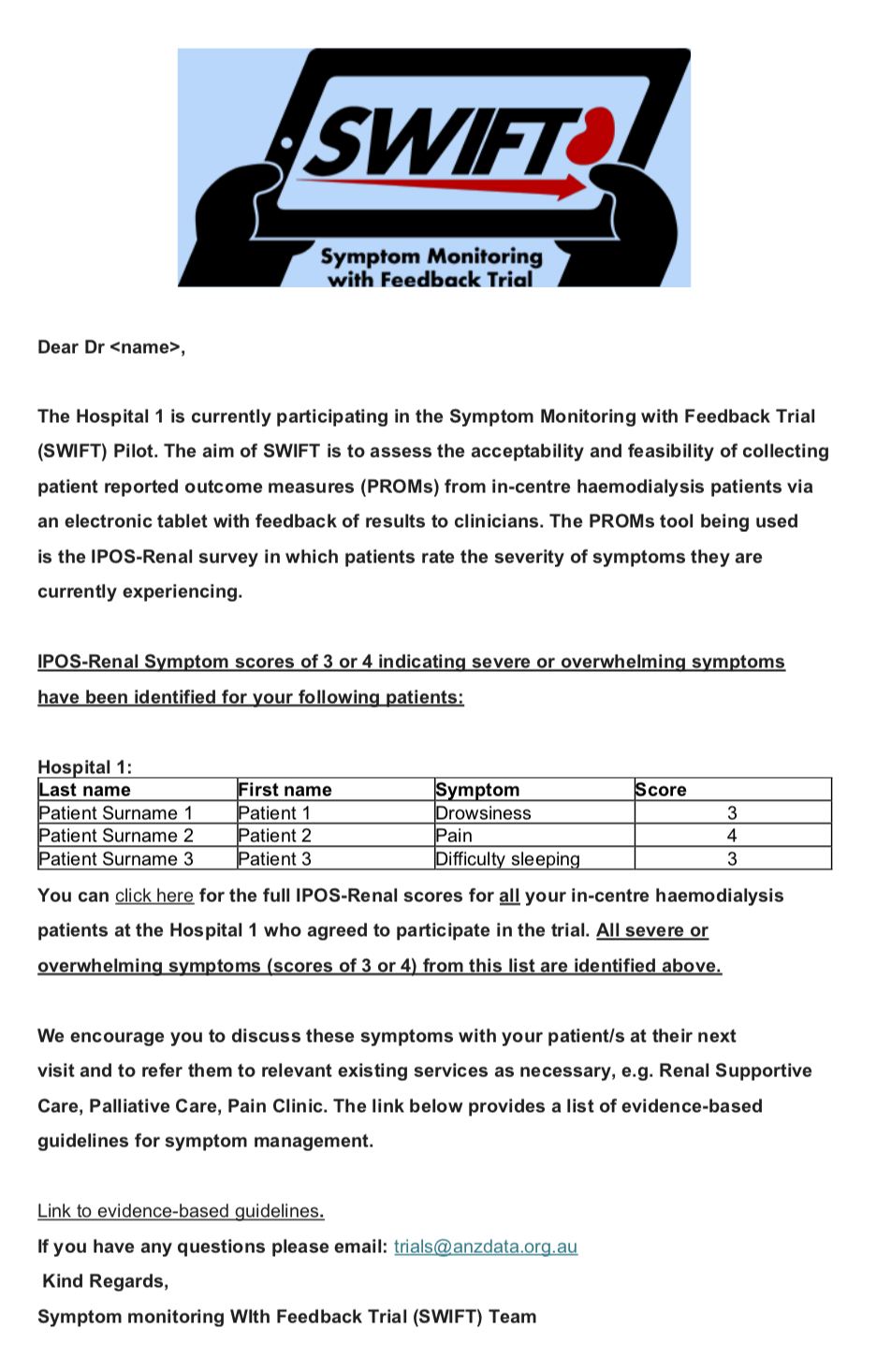
**

**Item S5.** Characteristics of ‘complete cases’ (participants who completed all questionnaires) by study allocation.

| Variable | Control  (n = 60) | Intervention  (n = 44) |
| --- | --- | --- |
| Age (years), mean (SD) | 63 (15) | 66 (13) |
| Sex   - Males, n (%) - Females, n (%) | 37 (62)  23 (38) | 34 (77)  10 (23) |
| Ethnicity, n (%) |  |  |
| - White European | 41 (68) | 42 (95) |
| - Indigenous | 3 (5) | 2 (5) |
| - Other | 16 (27) | 0 (0) |
| Co-morbidities*, n (%) |  |  |
| - Diabetes mellitus | 25 (42) | 21 (48) |
| - Ischemic heart disease | 22 (20) | 15 (34) |
| - Cerebrovascular disease | 9 (15) | 4 (9) |
| - Peripheral vascular disease | 13 (22) | 8 (18) |
| - Chronic lung disease | 12 (20) | 10 (23) |
| Primary kidney disease, n (%) |  |  |
| - Diabetic Nephropathy | 15 (25) | 9 (20) |
| - Glomerulonephritis | 10 (17) | 11 (25) |
| - Hypertension | 10 (17) | 4 (9) |
| - Other | 25 (41) | 20 (46) |
| Years on dialysis, median (IQR) | 2.37 (1.21, 5.15) | 2.09 (1.4, 2.90) |

IQR, Interquartile range; SD, Standard deviation

* Participants were likely to have more than one co-morbidity

**Item S6.** Frequency of levels by dimensions for ‘some problems’ in the EQ-5D-5L questionnaire by study allocation at baseline and 6-months.

| **Level** | **Mobility** | | | | **Self-Care** | | | | **Usual activities** | | | | **Pain and discomfort** | | | | **Anxiety and depression** | | | |
| --- | --- | --- | --- | --- | --- | --- | --- | --- | --- | --- | --- | --- | --- | --- | --- | --- | --- | --- | --- | --- |
|  | C0 | C2 | I0 | I2 | C0 | C2 | I0 | I2 | C0 | C2 | I0 | I2 | C0 | C2 | I0 | I2 | C0 | C2 | I0 | I2 |
| 1 (no problems) | 25 | 26 | 13 | 20 | 42 | 41 | 34 | 36 | 26 | 26 | 16 | 21 | 22 | 28 | 12 | 23 | 35 | 37 | 24 | 31 |
| 2 (slight problems) | 14 | 13 | 15 | 11 | 9 | 11 | 6 | 5 | 19 | 17 | 14 | 12 | 22 | 17 | 20 | 15 | 13 | 14 | 15 | 5 |
| 3 (moderate problems) | 15 | 15 | 7 | 7 | 9 | 8 | 3 | 3 | 8 | 15 | 9 | 7 | 11 | 11 | 8 | 5 | 11 | 7 | 3 | 7 |
| 4 (severe problems) | 5 | 6 | 6 | 2 | 0 | 0 | 1 | 0 | 3 | 2 | 2 | 1 | 3 | 3 | 4 | 0 | 1 | 2 | 1 | 0 |
| 5 (extreme problems/unable to do) | 1 | 0 | 3 | 4 | 0 | 0 | 0 | 0 | 4 | 0 | 3 | 3 | 2 | 1 | 0 | 1 | 0 | 0 | 1 | 1 |
| Total^a^ | 60 | 60 | 44 | 44 | 60 | 60 | 44 | 44 | 60 | 60 | 44 | 44 | 60 | 60 | 44 | 44 | 60 | 60 | 44 | 44 |
| Number of participants reporting some problem^b^ | 35  (58%) | 34  (57%) | 31  (70%) | 24  (55%) | 18  (30%) | 19  (32%) | 10  (23%) | 8  (18%) | 34  (57%) | 34  (57%) | 28  (64%) | 23  (52%) | 38  (63%) | 32  (54%) | 32  (73%) | 21  (48%) | 25  (42%) | 23  (38%) | 20  (45%) | 13  (30%) |
| Change in number of participants reporting some problem^b^ | -1 | | -7 | | +1 | | -2 | | 0 | | -5 | | -6 | | -11 | | -2 | | -7 | |
| % Change in number of participants reporting some problem^b^ from baseline | -3% | | -23% | | +6% | | -20% | | 0% | | -18% | | -16% | | -34% | | -8% | | -35% | |

C0 = Control arm at baseline, n=60; C2 = Control arm at 6-months, n=60; I0 = Intervention arm at baseline, n=44; I2 = Intervention arm at 6-months, n=44

^a^ Results are for those who responded at both baseline and subsequent questionnaires (‘complete cases’)

^b^ ‘Some problems’ = levels 2+3+4+5
